# Supplementary material for: The unfolded protein response sensor PERK mediates mechanical stress‐induced maturation of focal adhesion complexes in glioblastoma cells
Source: FEBS Lett. 2024 Aug 16;598(24):3021–35. doi: 10.1002/1873-3468.14996 (PMC11665954; doi:10.1002/1873-3468.14996)
Supplement: Supplementary file 1 — Fig. S1. Stiffness‐dependent F‐actin remodeling and differentiation are not affected by GSK414. Fig. S2. PERK‐deficient GG16 cells are impaired in cellular adaptation to increasing stiffness that is linked with aberrant FLNA expression. Fig. S3. Inhibition of F‐actin polymerization mimics phenotype of PERK‐deficient cells by impairing cellular adaption to matrix stiffness. Fig. S4. Effects of GSK414 and latrunculin B on talin and vinculin expression. Fig. S5. Effects of GSK414 and latrunculin B on tensin and integrin β1 expression. Fig. S6. Effects of GSK414 and latrunculin B on vimentin and tubulin expression. [file FEB2-598-3021-s001.pdf]

Supporting information

The unfolded protein response sensor PERK mediates mechanical stress-induced maturation of focal adhesion complexes in glioblastoma cells

Running Title: PERK regulates stiffness-dependent focal adhesion complex formation

Mohammad Khoonkari [1,2#], Dong Liang [1#], Marleen Kamperman [2], Patrick van Rijn [3,4], Frank A.E. Kruyt [1,*]

1- Department of Medical Oncology, University of Groningen, University Medical Center Groningen, Hanzeplein 1, 9713 GZ, Groningen, the Netherlands.
2- Zernike Institute for Advanced Materials, University of Groningen, Nijenborgh 4, 9747 AG Groningen, the Netherlands.
3- Department of Biomedical Engineering-FB40, University of Groningen, University Medical Center Groningen, A. Deusinglaan 1, 9713 AV Groningen, the Netherlands.
4- W.J. Kolff Institute for Biomedical Engineering and Materials Science-FB41, Groningen, University of Groningen, University Medical Center Groningen, A. Deusinglaan 1, 9713 AV Groningen, the Netherlands.

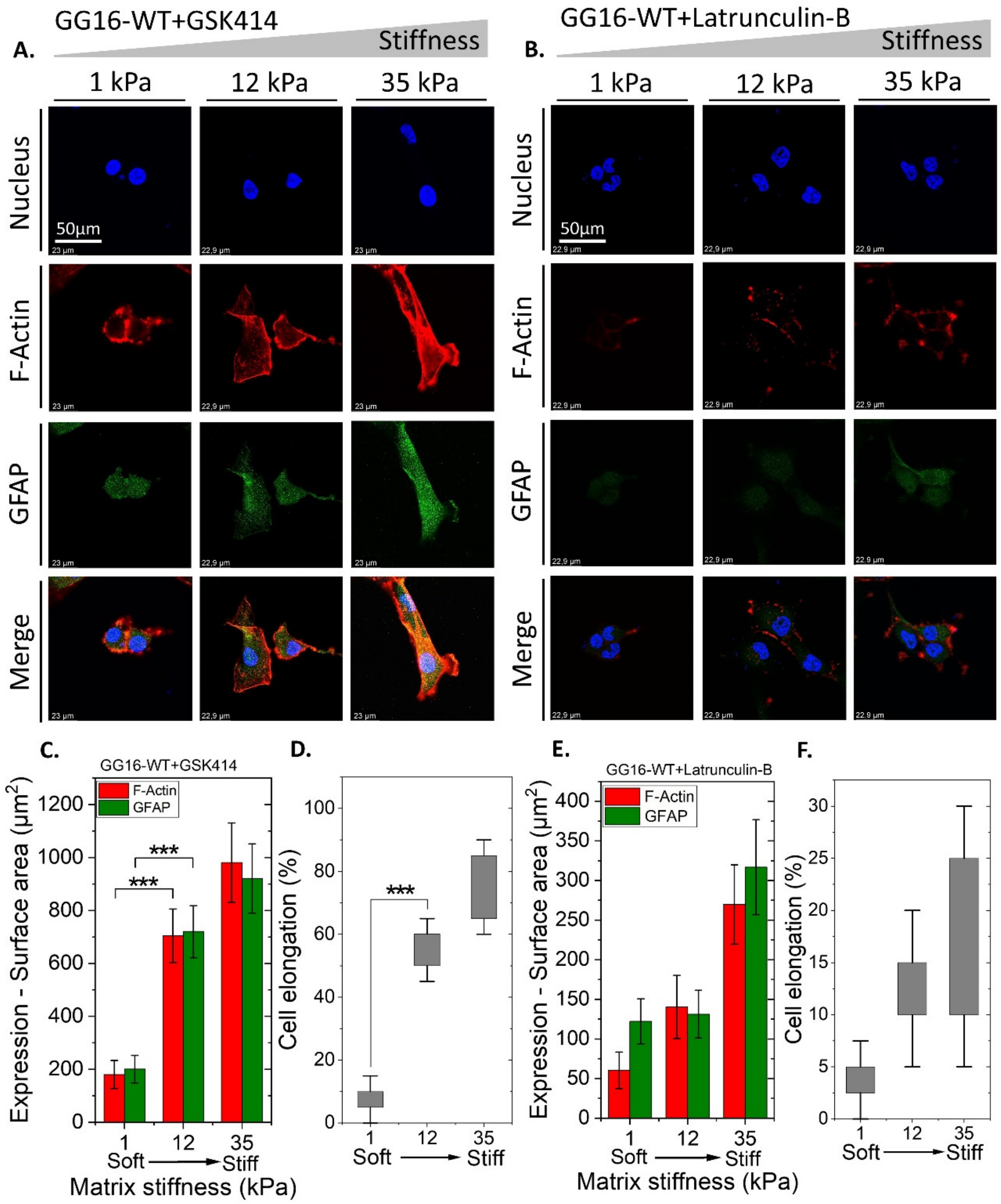

Figure S1. Stiffness dependent F-Actin remodeling and differentiation are not affected by GSK414. Confocal microscope images of GG16-WT cells treated with (A) GSK414 and (B) Latrunculin B and cultured on different matrix stiffnesses stained for F-Actin and GFAP. Expression levels (surface area of staining signals) measured for (C) GG16-WT+GSK414 and (E) GG16-WT+Latrunculin B. Cell elongation measured for (D) GG16-WT+GSK414 and (F) GG16-WT+Latrunculin B. Values are the mean ± SD. * $p \leq 0.05$; ** $p \leq 0.01$; *** $p \leq 0.001$.

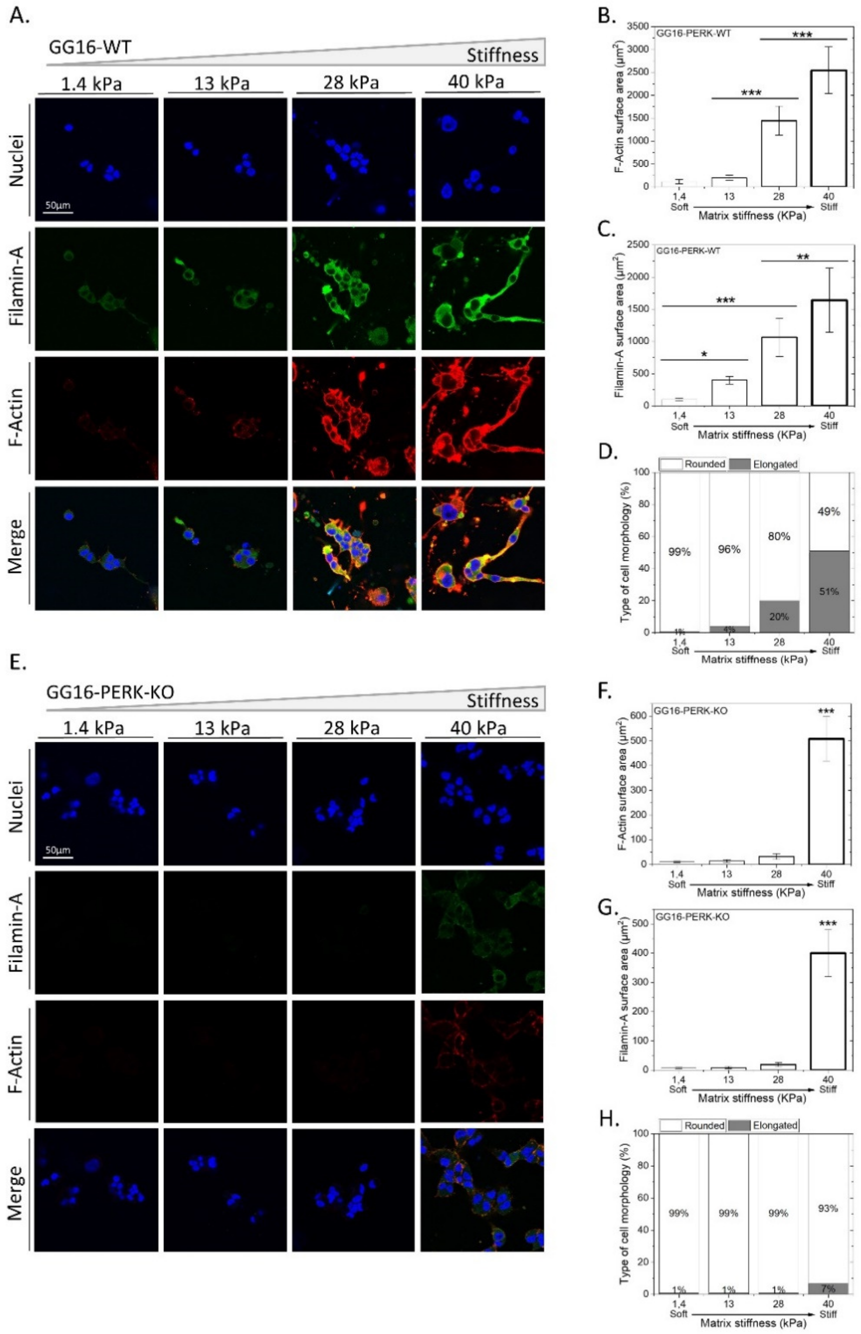

Figure S2. PERK deficient GG16 cells are impaired in cellular adaptation to increasing stiffness that is linked with aberrant FLNA expression. (A, E) GG16-WT and PERK-KO cells were cultured for 6 days on hydrogels with different stiffness and stained with DapiTM (Blue), Alexa fluorTM 546-Phalloidin (Red) and FLNA - Alexa fluorTM 488 (Green). Cell morphology, F-Actin and FLNA expression was quantified and is depicted in (B-D) and (F-H) for GG16-WT and PERK-KO cells, respectively. Cell morphology (from round to elongated), F-Actin and FLNA expression altered gradually in a stiffness-dependent manner in GG16-WT cells which was not seen in PERK deficient cells. Values are the mean ± SD. * $p \leq 0.05$; ** $p \leq 0.01$; *** $p \leq 0.001$.

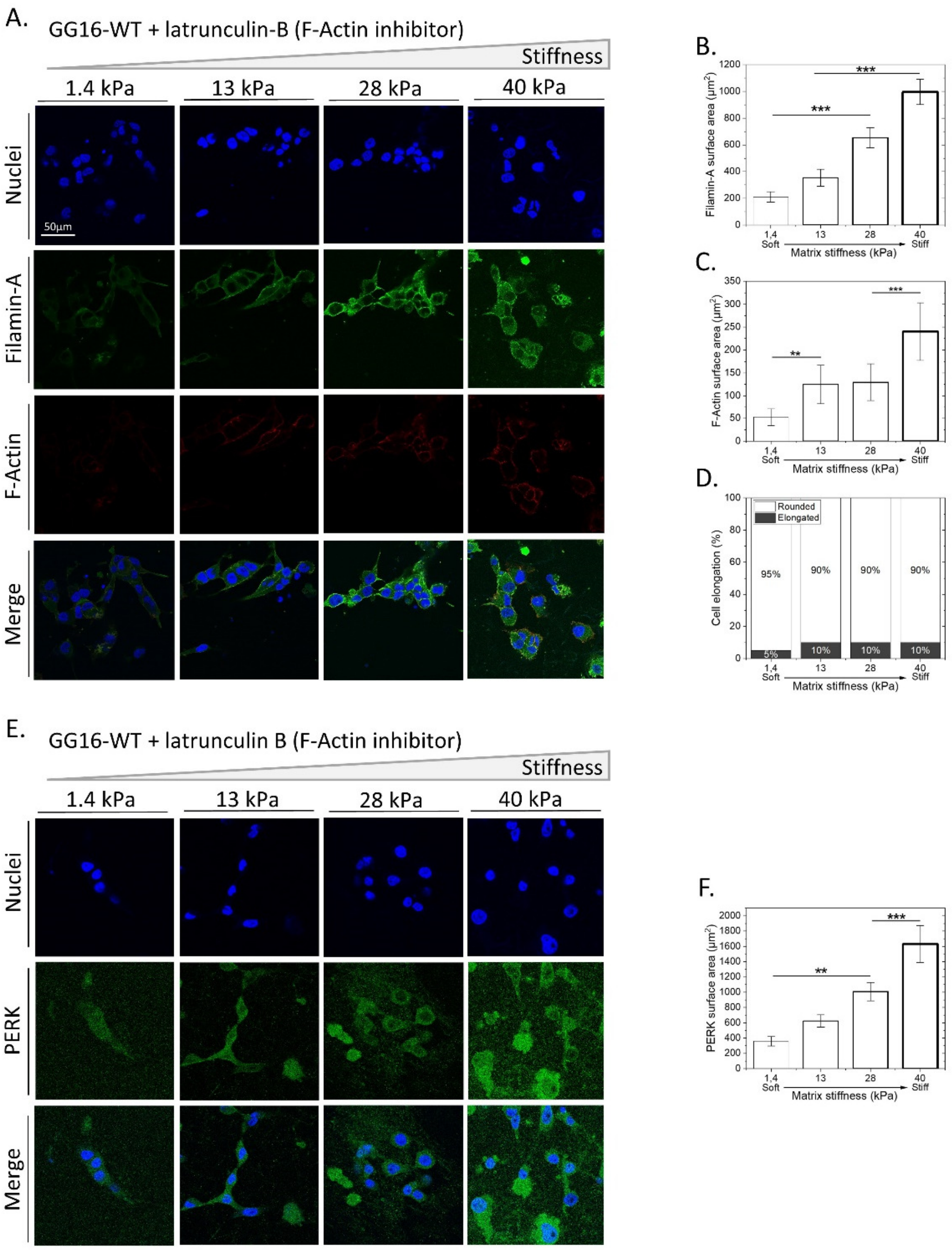

Figure S3. Inhibition of F-Actin polymerization mimics phenotype of PERK deficient cells by impairing cellular adaption to matrix stiffness. (A, E) GG16-WT cells treated with Latrunculin B were cultured for 6 days on hydrogels of varying stiffness. Cells were stained for F-Actin, FLNA and PERK and nuclei and imaged with confocal microscopy. Specific fluorescence was quantified for FLNA (B), F-Actin (C), cell elongation (D) and PERK expression (F). Values are the mean ± SD. * $p \leq 0.05$; ** $p \leq 0.01$; *** $p \leq 0.001$.

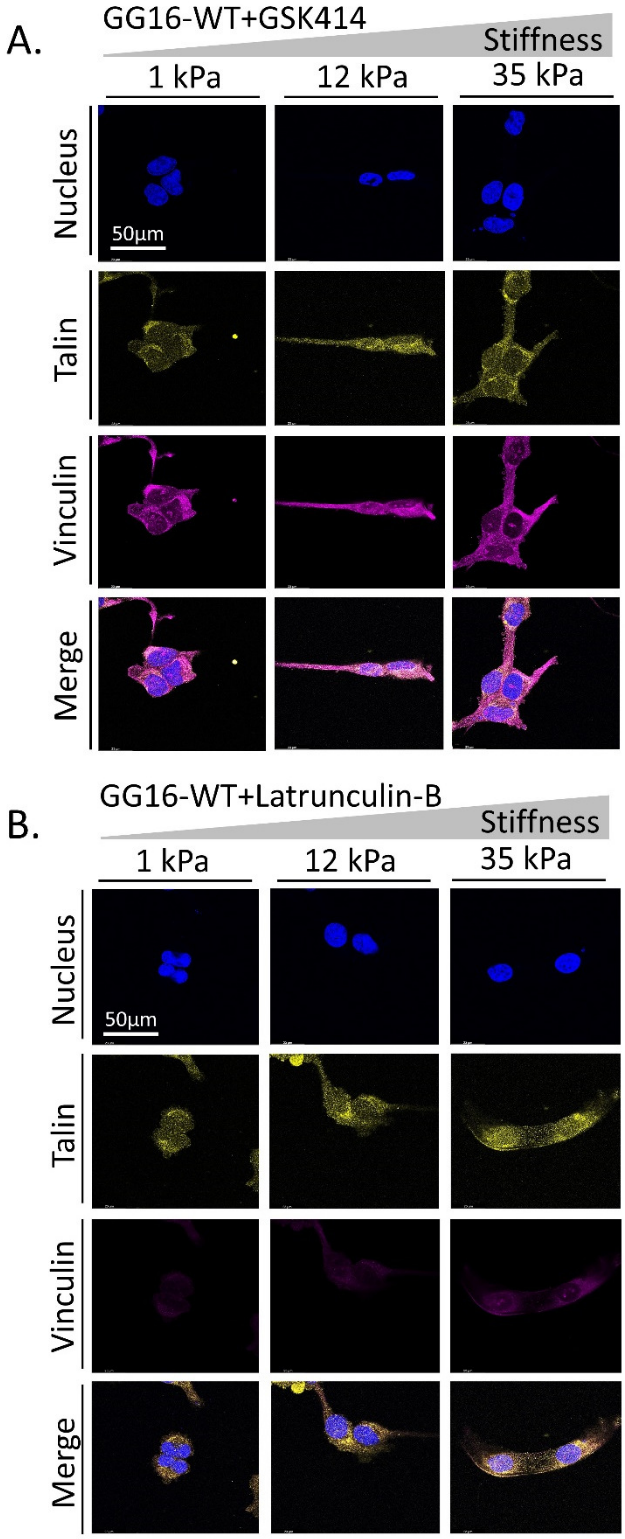

Figure S4. Effects of GSK414 and Latrunculin B on Talin and Vinculin expression. Confocal microscope images of GG16-WT cells treated with (A) GSK414 and (B) Latrunculin B, cultured on a hydrogels with indicated matrix stiffness and stained for Talin and Vinculin.

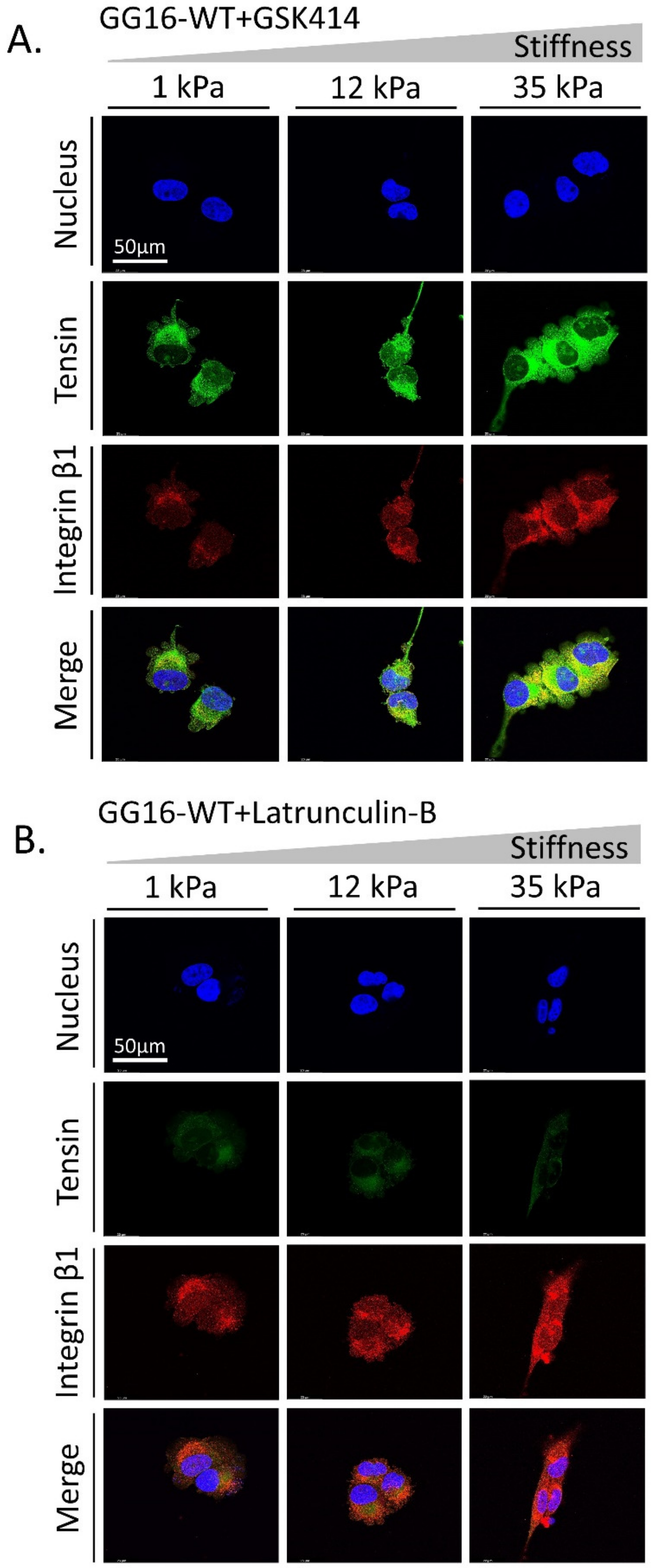

Figure S5. Effects of GSK414 and Latrunculin B on Tensin and Integrin β1 expression. Confocal microscope images of GG16-WT cells treated with (A) GSK414 and (B) Latrunculin B and cultured at different matrix stiffnesses and stained for Tensin and Integrin β1 expression.

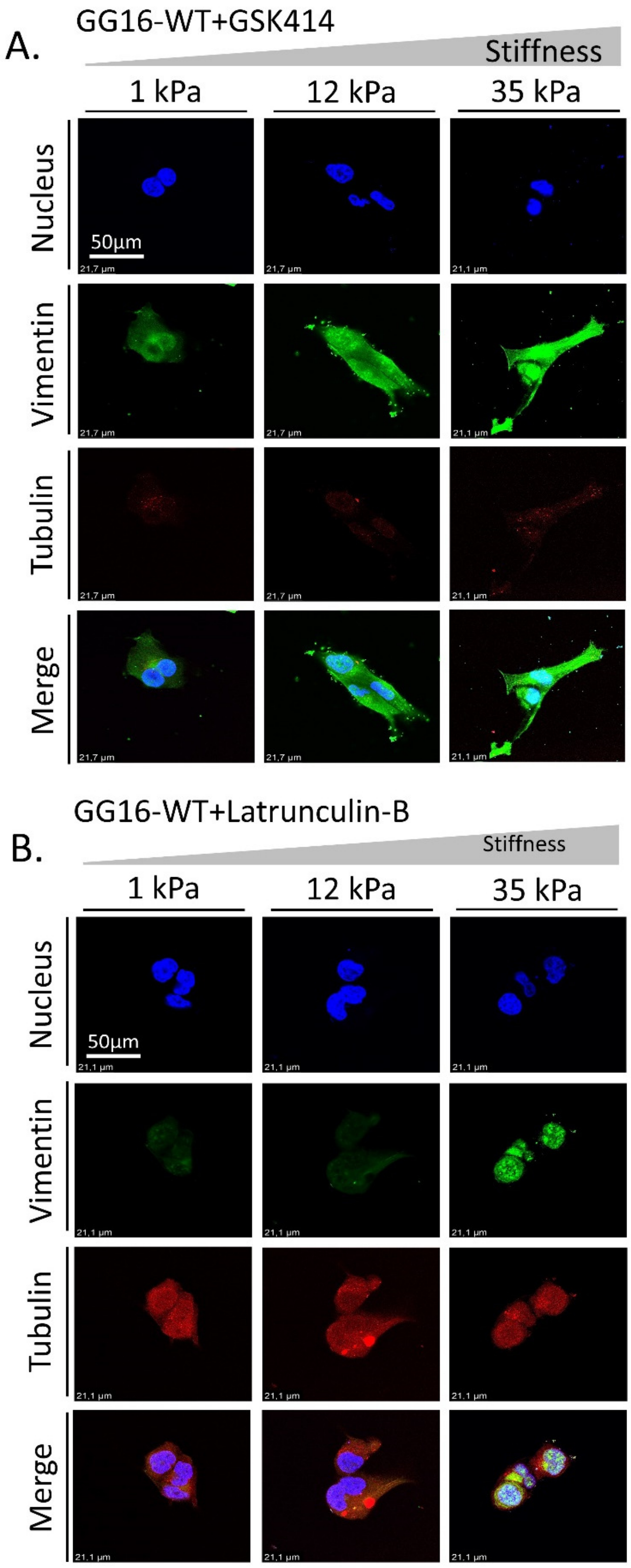

Figure S6. Effects of GSK414 and Latrunculin B on Vimentin and Tubulin expression. Confocal microscope images of GG16-WT cells treated with (A) GSK414 and (B) Latrunculin B and cultured on a range of matrix stiffnesses stained for Tubulin and Vimentin expression.
